# Mental health competencies are stronger determinants of well-being than mental disorder symptoms even in psychiatric samples

**DOI:** 10.1192/j.eurpsy.2024.150

**Published:** 2024-08-27

**Authors:** V. Zábó, D. Erát, A. Vargha, J. Harangozó, M. Iváncsics, Á. Vincze, J. Farkas, G. Balogh, R. Cowden, F. Pongrácz, J. Bognár, E. Nagy, G. Purebl, X. Gonda

**Affiliations:** ^1^Doctoral School of Psychology, Eötvös Loránd University; ^2^Institute of Psychology, Faculty of Education and Psychology, Eötvös Loránd University, Budapest; ^3^Institute of Social and Media Studies, Department of Sociology, University of Pécs, Pécs; ^4^Person- and Family-Oriented Health Science Research Group, Institute of Psychology, Faculty of Humanities and Social Sciences, Károli Gáspár University of the Reformed Church in Hungary; ^5^Community Psychiatry Centre, Semmelweis University – Awakenings Foundation; ^6^National Institute of Mental Health, Neurology, and Neurosurgery, Nyírő Gyula Hospital, Budapest, Hungary; ^7^Human Flourishing Program, Institute for Quantitative Social Science, Harvard University, Cambridge, United States; ^8^Institute of Behavioural Sciences, Faculty of Medicine, Semmelweis University; ^9^Department of Psychiatry and Psychotherapy, Semmelweis University; ^10^NAP3.0-SE Neuropsychopharmacology Research Group, Hungarian Brain Research Program, Semmelweis University, Budapest, Hungary

## Abstract

**Introduction:**

Exploring the positive psychological and behavioural dimensions of people living with mental disorders can establish a firm ground in a therapeutic alliance for setting up positive life goals.

**Objectives:**

The present study aimed to explored whether the strength of the mental health capacities and the severity of mental disorder symptoms and the interaction of the two differ in the strength of their associations with several dimensions of well-being on Hungarian adult psychiatric and non-clinical community samples.

**Methods:**

The psychiatric sample (129 patients (44 male, 85 female)) was collected in four Hungarian healthcare facilities using a cross-sectional design. The non-clinical community sample (253 adults (43 male, 210 female)) was collected online using a cross-sectional design. All the respondents completed the Mental Health Test, six well-being and mental health measures, and the Symptom Checklist-90-Revised.

**Results:**

Including both the mental health competencies and mental disorder symptoms variables in one regression model in both samples can predict patients’ well-being even more accurately. Mental health competencies related positively; mental disorder symptoms connected negatively to subjective well-being. In all models and both samples, mental health competencies were found to be a stronger determinant of well-being than the mental disorder symptoms. The interaction of mental health functioning and mental disorders is no more predictive of well-being in either psychiatric or non-clinical samples than when the effects of each are considered separately.

**Conclusions:**

The assessment of mental health competencies has an important predictive value for well-being in the presence of psychopathological symptoms and/or mental disorders.

**Disclosure of Interest:**

None Declared

